# CULTURALLY SENSITIVE STRATEGIES TO RECRUIT FOREIGN EDUCATED NURSES FOR US LONG-TERM CARE RESEARCH

**DOI:** 10.1093/geroni/igad104.3256

**Published:** 2023-12-21

**Authors:** Roy Thompson, Deidre Wipke-Tevis, Sherif Olanrewaju, Olumayowa A Odemuyiwa, Allison Squires

**Affiliations:** University of Missouri, Columbia, Missouri, United States; University of Missouri, Columbia, Missouri, United States; The Pennsylvania State University, University Park, Pennsylvania, United States; University of Missouri, Columbia, Missouri, United States; New York University, New York City, New York, United States

## Abstract

Long-term care (LTC) facilities experience chronic nursing staff instability which negatively impacts resident health outcomes. Recruiting Foreign Educated Nurses (FENs) is one strategy to stabilize the nursing workforce but most studies on FENs, use secondary data from: American Community Survey and the National Sample Survey of Registered Nurses. Few empirical studies have examined FEN experiences, employment outcomes, and impact on US LTC facilities. Since LTC facilities are dependent on FENs to meet minimum staffing requirements, additional research is needed to better understand FEN workforce issues in LTC, how to improve its sustainability, and the links to resident outcomes. Mistrust of researchers, however, may make FENs hard to reach. Therefore, conducting primary research on FENs in LTC requires culturally sensitive and community-engaged recruitment strategies. Drawing from existing literature and lessons learned from primary research conducted with FENs working in LTC facilities in the southeastern US, we will review best practices for recruitment so researchers can understand the cultural and structural issues that may affect research conduct in facilities where FENs are employed and their recruitment as participants. Developing relationships with FEN leaders as well as the Nigerian, Jamaican, Filipino, and/or Asian nurses associations can facilitate access to the population. Key stakeholder relationships also include the American Nurses Association, State Boards of Nursing, and Commission on Graduates of Foreign Nursing Schools as they can facilitate entrée into data sources and networks for recruitment. Understanding recruitment strategies will strengthen research that deepens our understanding of workforce issues among FENs in LTC.

